# Bioorthogonal non-canonical amino acid tagging to track transplanted human induced pluripotent stem cell-specific proteome

**DOI:** 10.1186/s13287-024-03792-3

**Published:** 2024-06-26

**Authors:** Divya Sridharan, Julie A. Dougherty, Uzair Ahmed, Shridhar K. Sanghvi, Syed Baseeruddin Alvi, Ki Ho Park, Helena Islam, Sue E. Knoblaugh, Harpreet Singh, Elizabeth D. Kirby, Mahmood Khan

**Affiliations:** 1 Division of Basic and Translational Sciences, Department of Emergency Medicine, College of Medicine, The Ohio State University, Columbus, OH 43210 USA; 2 Department of Physiology and Cell Biology, The Ohio State University, Columbus, OH USA; 3 Department of Molecular, Cellular and Developmental Biology, The Ohio State University, Columbus, OH USA; 4 Department of Surgery, University of Virginia, Charlottesville, VA USA; 5 Department of Veterinary Biosciences, The Ohio State University, Columbus, OH USA; 6 Department of Psychology, The Ohio State University, Columbus, OH USA; 7 Chronic Brain Injury Program, The Ohio State University, Columbus, OH USA

**Keywords:** Biorthogonal non-canonical amino acid tagging, Human induced pluripotent stem cells, Click chemistry, Cell-specific proteome, Paracrine signaling, Cell transplantation

## Abstract

**Background:**

Human induced pluripotent stem cells (hiPSCs) and their differentiated cell types have a great potential for tissue repair and regeneration. While the primary focus of using hiPSCs has historically been to regenerate damaged tissue, emerging studies have shown a more potent effect of hiPSC-derived paracrine factors on tissue regeneration. However, the precise contents of the transplanted hiPSC-derived cell secretome are ambiguous. This is mainly due to the lack of tools to distinguish cell-specific secretome from host-derived proteins in a complex tissue microenvironment in vivo.

**Methods:**

In this study, we present the generation and characterization of a novel hiPSC line, L274G-hiPSC, expressing the murine mutant methionyl-tRNA synthetase, L274G*Mm*MetRS, which can be used for tracking the cell specific proteome via biorthogonal non-canonical amino acid tagging (BONCAT). We assessed the trilineage differentiation potential of the L274G-hiPSCs in vitro and in vivo. Furthermore, we assessed the cell-specific proteome labelling in the L274G-hiPSC derived cardiomyocytes (L274G-hiPSC-CMs) in vitro following co-culture with wild type human umbilical vein derived endothelial cells and in vivo post transplantation in murine hearts.

**Results:**

We demonstrated that the L274G-hiPSCs exhibit typical hiPSC characteristics and that we can efficiently track the cell-specific proteome in their differentiated progenies belonging to the three germ lineages, including L274G-hiPSC-CMs. Finally, we demonstrated cell-specific BONCAT in transplanted L274G-hiPSC-CMs.

**Conclusion:**

The novel L274G-hiPSC line can be used to study the cell-specific proteome of hiPSCs in vitro and in vivo, to delineate mechanisms underlying hiPSC-based cell therapies for a variety of regenerative medicine applications.

**Supplementary Information:**

The online version contains supplementary material available at 10.1186/s13287-024-03792-3.

## Introduction

Human induced pluripotent stem cells (hiPSCs) have revolutionized the field of regenerative medicine in the last ten years [1]. The ability to develop patient-derived hiPSCs have opened new avenues to tailor personalized medicine approaches for numerous disorders and degenerative diseases [2]. Furthermore, the expandability and pluripotency of hiPSCs has been critical for their use in developing cell-based therapies for traumatic brain injury [3, 4], spinal cord injury [5, 6], myocardial infarction [7, 8], acute kidney injury [9], and diabetes mellitus [10, 11]. However, despite the improved functional outcomes observed in preclinical studies involving hiPSC-based cell therapy, several studies reported poor engraftment and long-term survival of the transplanted cells [12–14].

Emerging studies have attributed the beneficial effect of hiPSC-based cell therapies on tissue repair and regeneration to the paracrine factors derived from these cells [12–14]. The hiPSC secretome containing bioactive growth factors, proteins, nucleic acids, and extracellular vesicles has been extensively studied for their therapeutic potential in recent years [15]. Despite their therapeutic benefits, the precise composition of the transplanted hiPSC secretome in vivo has not been established. While the extracellular microenvironment and interaction with other cell-types (in complex tissues) have been known to modulate the stem cell secretome, most studies have characterized the stem cell secretome in vitro using single cell-type cultures [16, 17]. The main challenge in studying stem cell secretome in vivo or in multi-cell-type cultures is the unavailability of tools to track cell-specific proteome.

In the present study, we adapted a novel technique for protein labeling, known as bioorthogonal non-canonical amino acid tagging (BONCAT), to identify the hiPSC-specific proteome. We expressed a mutant mouse methionyl tRNA synthetase (L274G*Mm*MetRS, hereafter “L274G”) in hiPSCs. The mutation in L274G facilitates the competitive charging of methionyl-tRNA with a non-canonical amino acid, azidonorleucine (Anl), instead of the endogenous methionine. Culturing L274G-expressing hiPSCs (L274G-hiPSCs) in the presence of Anl enabled us to create a cell-specific protein tag in these cells which could be detected by click chemistry between a biologically rare azide in Anl and an alkyne, Dibenzocyclooctyne (DBCO)-IR800. These alkyne-conjugated, Anl-tagged proteins were then detected and characterized by polyacrylamide gel electrophoresis. Using this technique, we successfully detected the proteins made and secreted by the L274G-hiPSC-CMs both in a co-culture with wild type human umbilical cord endothelial cells (HUVECs), as well as post-transplantation into an animal model in vivo.

Taken together, our study is the first to establish and characterize a hiPSC line expressing the new and innovative BONCAT technology to understand the mechanisms underlying stem-cell mediated tissue regeneration in various disease pathologies like traumatic brain injury, myocardial infarction, and diabetes mellitus.

## Materials and methods

All experiments have been performed and reported in accordance with the ARRIVE guidelines 2.0.

### Lentivirus production

The L274G-T2A-mCherry sequence from Mahdavi et al. [18] was synthesized by Genscript Inc and inserted into a lentiviral backbone (pLJM1-EGFP, Addgene #19319) [19]. EF1α promoter sequence was also synthesized by Genscript and cloned to replace the CMV promoter. Plasmids were grown in One Shot Stbl3 chemically competent cells (Invitrogen, #C737303) and prepared using a Qiagen HiSpeed Maxi prep kit (Qiagen, #12662). Plasmid identity was confirmed via restriction digestion and Sanger sequencing (EF1a F 5’-TCAAGCCTCAGACAGTGGTTC; mCherry R 5’-TTGGTCACCTTCAGCTTGG, both from Addgene, synthesized by IDT DNA). VSV-G pseudotyped 3rd generation lentiviral particles were prepared by Vigene Biosciences Inc.

### Lentiviral transduction of hiPSCs

#### Passaging and maintenance of hiPSCs

CYS0105 hiPSCs (ATCC, henceforth WT-hiPSCs), reprogrammed from cardiac fibroblasts were procured and cultured as described previously [20]. Briefly, the cryopreserved hiPSCs were thawed and cultured in Essential 8™ Medium (E8, Thermo Fisher Scientific, MA) on Cell Basement Membrane (ATCC)-coated 6-well plates (Fisher Scientific). For the first 24 h, the culture medium was supplemented with 10 μM Y-27632 (TOCRIS, MN). The cultures were passaged using Gentle Cell Dissociation Reagent (Stem Cell Technologies) at a confluency of > 80% onto Cell Basement Membrane coated dishes as previously described [20].

#### Lentiviral transduction

*Day 0:* The WT-hiPSCs were passaged at 1:6 ratio on to Cell Basement Membrane-coated dishes in E8 medium supplemented with 10 µM Y27632.

*Day 1:* The medium was replaced with 2 ml/well of prewarmed E8 medium supplemented with 6 µg/ml polybrene (Millipore) and cultures were incubated at 37 °C, 5% CO_2_ for 15 min. To each well, 10 µl of 1 × 10^6^ TU/ml of L274G lentiviral particles were added and incubated at 37 °C, 5% CO_2_ for 24 h.

*Day 2:* The medium was replaced with 2 ml/well of prewarmed E8 medium supplemented with 6 µg/ml polybrene (Millipore) for 15 min followed by addition of 30 µl of 1 × 10^6^ TU/ml of L274G lentiviral particles.

*Day 3 and 4:* The medium was replaced with fresh E8 medium. mCherry fluorescence was visible in the transduced hiPSCs by the end of Day 4.

*Day 5–10 (Puromycin Selection):* E8 medium supplemented with 1 µg/ml puromycin was added every day to the cultures.

The selected hiPSCs were passaged and maintained as described previously [20].

### Trilineage differentiation of L274G-hiPSCs

Trilineage differentiation potential of the WT-hiPSCs and L274G-hiPSCs was assessed using the STEMdiff™ Trilineage Differentiation Assay kit (Stem cell Technologies) per the manufacturer’s directions. Briefly, the hiPSCs were passaged at seeding densities of 800,000/well (ectoderm and endoderm) and 200,000/well (mesoderm) in E8 medium supplemented with 10 µM Y-27632 in Cell Basement Membrane-coated 12-well plates. After 24 h, the medium was replaced with the appropriate trilineage differentiation medium (ectoderm, endoderm and mesoderm). Medium was replaced every 24 h for 4 days (mesoderm and endoderm) and 6 days (ectoderm), respectively.

### Cardiac differentiation of hiPSCs

WT-hiPSCs and L274G-hiPSCs were differentiated to functional cardiomyocytes as described previously [21]. Briefly, the hiPSCs were cultured in E8 medium until they reached a confluence of 90%. The hiPSCs were treated with Cardiac Differentiation medium I (CDM-I; RPMI medium supplemented with B27 supplement minus insulin) supplemented with 10 µM CHIR99021 for 24 h. The following day, the medium was replaced with CDM-I for 48 h. On day 3, CDM-I supplemented with 5 µM IWP-4 was added to the cultures and left for two days after which CDM-I was changed every alternate day until the cells started contracting spontaneously (~ 9 to 10 days). Once the cells start contracting, the medium was switched to cardiac differentiation medium II (CDM-II, RPMI supplemented with B27 supplement) for two days. The spontaneously contracting hiPSC-CMs were enriched in culture by metabolic selection in selection medium (DMEM without glucose supplemented with 1% non-essential amino acids, 1% GlutaMax, 1% penicillin–streptomycin, and 4 mM L-lactate) as previously described [21]. Post-enrichment the hiPSC-CMs were trypsinized, re-plated on to Cell Basement Membrane-coated 6-well plates in cardiomyocyte maintenance medium (CMM; CDM-II supplemented with 2.5% fetal bovine serum, FBS). The medium was changed every alternate day.

### Co-culture of hiPSC-CMs and human umbilical vein endothelial cells (HUVECs)

Differentiated L274G-hiPSCs were co-cultured with wild type HUVECs to validate cell-specific protein labelling in vitro. HUVECs were procured from Lonza, and the cells were cultured and maintained per the manufacturer’s recommendations. Briefly, the HUVECs were thawed and cultured in Clonetics™ EGM™-2 BulletKit™ (Lonza Catalog No. CC-3162) which comprises of Endothelial Basal Medium-2 (EBM™-2 Medium) supplemented with human Epidermal Growth Factor (hEGF), Vascular Endothelial Growth Factor (VEGF), R3-Insulin-like Growth Factor-1 (R3-IGF-1), Ascorbic Acid, Hydrocortisone, human Fibroblast Growth Factor-Beta (hFGF-β), Heparin, 2% FBS, and Gentamicin/Amphotericin-B. For co-culture, HUVECs and L274G-hiPSCs were dissociated using 0.25% trypsin–EDTA for 3 min and 7 min, respectively. The trypsin activity was neutralized by adding equal volume of their respective culture medium containing FBS. The cell suspensions were centrifuged, and cell pellets were re-suspended in 5 ml of HUVEC culture medium and CMM mixed at a ratio of 1:1. The cell count for each cell-type was determined using the Countess 3 automated cell counter (Thermofisher Scientific). The L274G-hiPSC-CMs and HUVECs were mixed at a ratio of 3:1 and co-cultured for up to 5 days in HUVEC culture medium: CMM (1:1).

### Teratoma formation assay

L274G-hiPSCs (p15) and WT-hiPSCs (p22) were dissociated into a single cell suspension using Gentle Cell Dissociation Reagent and resuspended in E8 medium for cell count. Approximately 1.5 million L274G-hiPSCs were resuspended in 50 µl Matrigel, and injected subcutaneously into the right flank of six-week-old immunocompromised female (NOD.CB17*-Prkdc*^*scid*^/NCrCrl, Charles River*)* mice (n = 5), as previously described [22]. As a control, an equal number of WT-hiPSCs were injected into the left flank of the same mice. The mice were anaesthetized using 1.5% isoflurane during the procedure. The mice were allowed to recover, and we monitored twice a week till they developed teratomas (~ 6 to 8 weeks). The mice were humanely euthanized by carbon dioxide exposure once teratomas reached a diameter of 2 cm. The subcutaneous teratomas were surgically excised, fixed in 10% neutral buffered formalin, and embedded in paraffin. Paraffin sections of 4 µm were stained with hematoxylin and eosin (H&E) and evaluated by light microscopy (Nikon Eclipse Ci, Nikon Instruments, Melville, NY) with attached SC50 digital camera, (Olympus, B and B Microscopes Limited, Pittsburgh, PA) by a board-certified veterinary pathologist (S.E.K.).

### Transplantation of hiPSC-CMs to murine hearts

Immunocompromised female NOD SCID mice (NOD.CB17*-Prkdc*^*scid*^/NCrCrl, Charles River*)* were procured and housed in the University Laboratory Animal Resources (ULAR), The Ohio State University. Per previous publications [23], to access in vivo BONCAT in transplanted L274G-hiPSC-CMs, ten mice were randomly assigned into two groups: (a) Control and (b) cell transplant (n = 5/group). The mice in both the groups were fed a fixed 0.2% methionine (met) diet (Teklad Custom Diet, Envigo) for the duration of the study (two weeks). For the cell transplant group, the mice were anesthetized using isoflurane (1.5–3.0%), the chest cavity will be opened between 2nd and 3rd ribs and approximately 2.5 million L274G-hiPSC-CMs (suspended in 20 µl Matrigel) were intramyocardially delivered into the left ventricle. The chest incisions were closed with ~ 4-0 polypropylene/Ti-Cron sutures, and upon completion of the surgical procedures, all animals were closely monitored to confirm recovery from anesthesia (resume normal behavior, rhythmic breathing without assistance). The weight and health of the mice were monitored daily for the duration of the experiment to ensure the adequacy of their diet. Animals with a weight loss > 20% were excluded from the study. Daily intraperitoneal (IP) injections of Anl (0.2 mmol/kg body weight) were given to both the control and cell transplant groups for five days before the end of the study. At two weeks, the mice were euthanized, the plasma, liver, kidney, lungs, and hearts were collected, and frozen at − 80 °C.

### Immunostaining

For immunocytochemical analysis, cells were cultured and/or differentiated on glass coverslips as previously described [20]. The cells were fixed using 4% paraformaldehyde for 15 min at RT and permeabilized using 0.2% triton X-100 for 5 min on ice. Non-specific antibody binding was blocked by incubating the cells in blocking buffer containing 1% bovine serum albumin (BSA) in PBS for 1 h at RT. The cells were incubated with primary antibodies diluted in blocking solution at 4 °C, overnight with gentle shaking. The following day, the cells were washed thrice in PBS and incubated with the corresponding secondary antibody diluted in blocking buffer for 1 h at RT in dark. The cell nuclei were then counterstained with DAPI for 15 min, at RT in dark. Finally, the coverslips were mounted on to glass slides using Prolong Gold Antifade. All incubation steps were followed by three washes with PBS. The following antibodies were used: anti-OCT4 (A24867, 1:200, Invitrogen), anti-SSEA4 (A24866, 1:200, Invitrogen), anti-SOX2 (A24759, 1:200, Invitrogen), anti-TRA-1-60 (A24868, 1:200, Invitrogen), anti-Nestin (A11861, 1:500, Abclonal), anti-SOX17 (AF1924, 1:500, R&D systems), anti-BryT (ab209665, 1:200, Abcam), anti-α-sarcomeric actinin (A7732, 1:500, Sigma-Aldrich), anti-troponin T (HPA017888, 1:500, Sigma-Aldrich), anti-mCherry (ab183628, 1:500, Abcam), goat anti-mouse AlexaFluor™ 488 (A11001, 1:1000, Invitrogen), goat anti-rabbit AlexaFluor™ 488 (A78953, 1:1000, Invitrogen), goat anti-mouse AlexaFluor™ 594 (A11005, 1:1000, Invitrogen), goat anti-rabbit AlexaFluor™ 594 (A11012, 1:1000, Invitrogen), donkey anti-goat AlexaFluor™ 488 (A11055, 1:1000, Invitrogen), and goat anti-rat AlexaFluor™ 488 (A11006, 1:1000, Invitrogen).

### Flow cytometry

The hiPSC cultures were washed twice in PBS and dissociated to single cells as described above. The viable cell number was determined using a hemocytometer after staining the cells with 0.4% trypan blue dye. The cells were fixed in 4% PFA for 15 min at RT. The cell suspension was centrifuged and the cell pellet was washed thrice with PBS. For permeabilization, the cell pellet was resuspended in 0.2% triton X-100 in PBS for 5 min on ice. The cells were again washed thrice in PBS and the cell pellet was resuspended in 1% BSA solution for blocking at a density of 5 × 10^6^ cells/ml and incubated for 1 h on ice. The cells (~ 1 million/200 µl) were then stained with the primary antibody (diluted in 1% BSA) for 1 h on ice. The cells were then washed thrice with PBS and incubated with the corresponding secondary antibodies for 30 min on ice. Finally, the cells were washed thrice in PBS and resuspended in 400 µl of PBS for analysis. Flow cytometry experiments were performed using BD LSRFortessa system (BD Biosciences) the data was analyzed by using the FlowJo software. The details of the antibodies used are provided in section "Immunostaining".

### Non-canonical amino acid tagging

BONCAT labelling was performed as previously described [24]. Briefly, undifferentiated, or differentiated hiPSC cultures were washed twice with phosphate-buffered saline (PBS) and incubated in methionine-depleted medium for 30 min at 37 °C, 5% CO_2_ to wash off residual methionine from the cultures. Medium containing varying concentrations (0–100 µM) of methionine and (0–2 mM) Anl was added to the cells and the cultures were incubated overnight at 37 °C, 5% CO_2_. The following day, the medium was replaced with regular culture medium, and the cultures were incubated for an hour, 37 °C, 5% CO_2_ to wash off the free Anl from the medium.

### Fluorescent non-canonical amino acid tagging (FUNCAT)

In vitro cultured hiPSCs or their derived differentiated cells were fixed in ice-cold methanol for 10 min at 4 °C after three washes with PBS. The methanol was washed off from the cells with PBS, and the cells were incubated in 20 mM iodoacetamide (IAA, Roche) for 1.5 h, at RT in dark. Aza-dibenzocyclooctyne (DBCO)-FITC was spiked in, to attain a final concentration of 12 µM and the cells were incubated for 10 min in dark at RT. The unbound DBCO-FITC was thoroughly washed off from the cells by three washes with PBS for 5 min each, with gentle shaking. Further, the cells were immunostained with specific antibodies as described above.

For FUNCAT immunohistochemical analysis, the L274G-hiPSC-CM-transplanted hearts were perfused and washed in PBS to remove blood. The tissue was embedded in OCT and cryosectioned as previously described. The cryosections were fixed and permeabilized by incubating with ice-cold methanol for 10 min on ice. The methanol was washed off with PBS and the cryosections were further incubated with 20 mM IAA for 2 h at RT in dark in a humidified chamber. DBCO-FITC was spiked in to achieve a final concentration of 12 µM and the sections were incubated overnight at RT in dark. The following day, the DBCO-FITC was thoroughly washed off with three PBS washes and the sections were incubated with blocking buffer containing 10% normal goat serum (NGS) in PBS for 1 h in dark. The sections were then incubated with anti-mCherry antibody diluted in the blocking buffer (1:200), overnight at 4 °C in dark. The next day the sections were washed in PBS and incubated anti-rabbit AlexaFluor™ 594 antibody diluted in the blocking buffer (1:1000) for 1 h at RT in dark. The sections were then counterstained with DAPI and imaged on the Olympus FV3000 microscope.

### BONCAT

To identify BONCAT of cellular proteins, hiPSCs were cultured in a 96-well plate, fixed in ice cold-methanol, and incubated with IAA as described above. After IAA incubation, DBCO-IR800 was spiked-in, and the cells were incubated for 10 min in dark at RT. The DBCO-IR800 was washed off with PBS, and the cells were dried completely at RT and imaged on the LICOR CLx system at 800 nm. Total protein in the cells was assessed by staining the cells with Page Blue and imaging at 700 nm using the LICOR CLx system. The signal intensities in the captured images were analyzed using the Image J software.

For western blotting, cell pellets or 30 mg of mouse left ventricles were resuspended in 8 M Urea (Sigma-Aldrich) containing protease inhibitors (Roche) and lysed by sonication (10% amplitude, 3 cycles of 1 s) on ice. The lysates were centrifuged, and supernatants were stored at − 80 °C until use. The protein concentration was assessed using the calorimetric Braford’s assay as previously described. BONCAT was performed as previously described. Briefly, 30–50 µg of protein was incubated with 20 mM IAA for 1.5 h in dark. DBCO-IR800 (final concentration of 12 µM) was spiked into the reaction mixture and incubated for an additional 30 min in dark. 1× Lamelli buffer containing β-mercaptoethanol was added and the samples were boiled at 95 °C for 10 min. The samples were resolved on a 4–20% gel and transferred onto nitrocellulose membranes. The membranes were blocked with 5% non-fat dry milk in TBS (NFDM, Biorad) and stained with primary antibodies diluted in 1% NFDM overnight at 4 °C on a shaker. The following day the membranes were stained with the corresponding HRP-conjugated secondary antibodies, developed using the Immobilon® UltraPlus Western HRP Substrate (EMD Millipore) per the manufacturer’s instructions, and imaged in the Azure C600 imaging system. Total protein loaded in the gel was assessed by staining the blots with Ponceau S stain. The secondary antibodies used were donkey anti-goat HRP (sc2020, 1:10,000, Santa Cruz Biotechnology), sheep anti-mouse HRP (NA931V, 1:10,000, GE Healthcare), and donkey anti-rabbit HRP (NA943V, 1:10,000, GE Healthcare).

L274G-hiPSC-CM-derived proteins in mouse plasma were identified as previously described [24, 25]. Briefly, 250 µl of mouse plasma was diluted (1:1) with 8 M urea containing protease inhibitors. Endogenous biotinylated proteins were eliminated by incubating the mouse plasma overnight with streptavidin-agarose beads followed by centrifugation. The supernatant was alkylated with 100 µM IAA for 2 h in dark and click reacted with 10 µM DBCO-biotin (Click Chemistry Tools) for 2 h in dark. The samples were then incubated overnight at 4 °C with the Human Growth Factor Array C1 (Ray Biotech). The following day, the arrays were washed and incubated with Streptavidin-HRP per the manufacturer’s instructions. The arrays were imaged as described above and signals were quantified using Image J. The signal intensities were normalized per the manufacturer’s (instructions, using the control mouse plasma as the reference array.

### Calcium imaging

L274G-hiPSC-CMs were cultured in CMM with or without supplementation with 0.125 mM Anl for one week. The L274G-hiPSC-CMs were then stained with Fluo-4AM and imaged on the Nikon A1R confocal microscope using the line scan mode to record the calcium transients as previously described [26]. The captured images were then analyzed using the Image J software as previously described [27].

### Statistical analysis

All values are presented as mean values ± standard error. The statistical significance between the groups was determined by Student’s t-test. For the mouse plasma samples, three arrays per group were analyzed using the manufacturer’s (Ray Biotech) instructions. One plasma sample from the control group was randomly assigned as the reference array. Finally, the fold-change of signal intensities in cell transplant group was calculated by normalizing with the control group, and significance was assessed using two-way ANOVA. Values were considered significant if the P values were < 0.05. All statistical analyses were performed using GraphPad Prism Software, version 10.

## Results

### Generation of L274G-hiPSCs

**Fig. 1:**
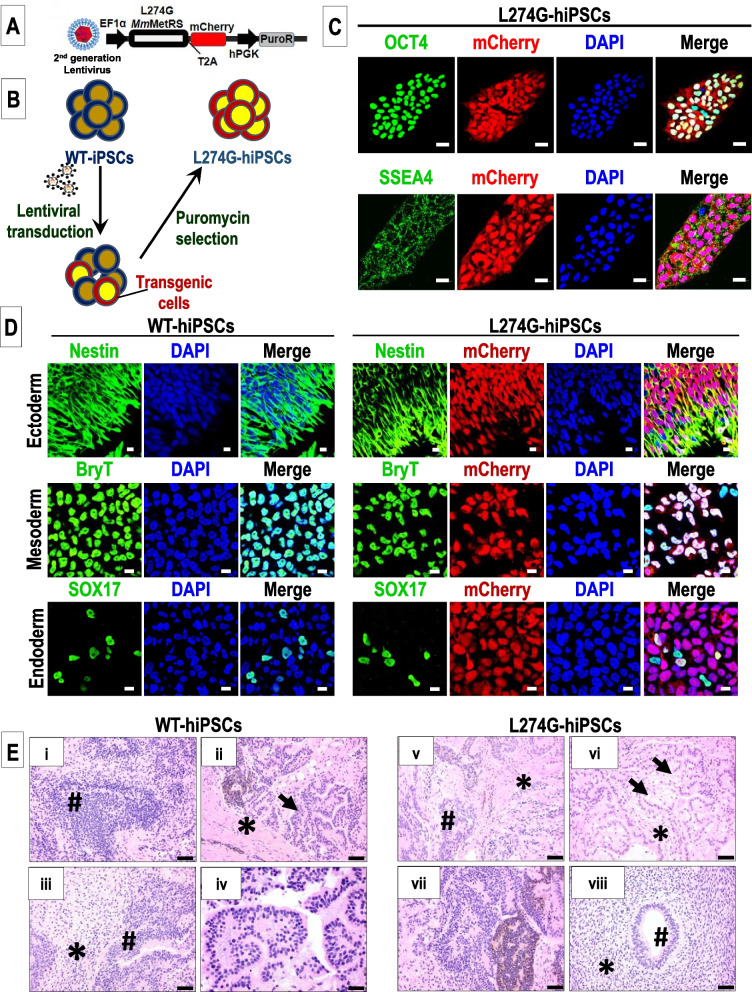
Generation and characterization of L274G-hiPSCs. **A** Lentivirus construct for expression of L274G in hiPSCs. **B** Experimental design for L274G lentiviral transduction of WT-hiPSCs. **C** Immunostaining for PSC markers, OCT4 and SSEA4 in L274G-hiPSCs. Scale bar: 20 µm. **D** Fluorescence images showing expression of Nestin, BryT, and SOX17 in in vitro differentiated L274G-hiPSCs and WT-hiPSCs. Scale bar: 20 µm. **E** Representative Hematoxylin and Eosin (H&E) stained images of hiPSC-derived teratomas showing (i) WT-hiPSC-derived immature neuroectoderm, (ii) WT-hiPSC-derived immature epithelium (endoderm, arrows) surrounded by bands of muscle (mesoderm, *), (iii) WT-hiPSC derived immature neuroectoderm (#) surrounded by primitive-appearing spindle cell stroma (mesoderm,*), (iv) WT-hiPSC-derived immature epithelium (endoderm). (v) L274G-hiPSC-derived immature neuroectoderm (#) separated by bands of muscle (mesoderm, *), (vi) L274G-hiPSC-derived primitive epithelium (endoderm, arrows) surrounded by bands of muscle (mesoderm, *), (vii) L274G-hiPSC-derived immature neuroectoderm, and (viii) L274G-hiPSC-derived neuroectodermal tubule (#) surrounded by primitive-appearing spindle cell stroma (mesoderm, *). Scale bar: (i-iii) and (v-viii): 50 µm; (iv): 20 µm

Third generation lentiviral particles carrying the L724G plasmid construct were generated for transduction (Fig. 1A). The L274G and mCherry transgenes were expressed under the constitutively active EF1α promoter. The two transgenes were separated by the T2A peptide sequence to enable cleavage of the two polypeptides. The puromycin resistance transgene was placed under the hPGK promoter. WT-hiPSCs were transduced with these lentiviral particles and mCherry fluorescence was monitored (Fig. 1B). We observed mCherry fluorescence in ~ 10% hiPSCs at 72 h after the secondary infection. The positively transduced, mCherry expressing L724G-hiPSCs were enriched by treatment with 1 µg/ml puromycin for five days (Fig. 1B). The enriched L274G-hiPSCs formed colonies typical of PSCs in culture and comparable with the WT-hiPSCs (Supplementary Fig. S1A). Both the L274G-hiPSCs and WT-hiPSCs expressed the pluripotency markers OCT4, SSEA4, SOX17 and TRA-1-60 as evidenced by the immunolocalization studies (Fig. 1C; Supplementary Fig. S1B, C). The stable integration of the transgene was confirmed using flow cytometry which showed mCherry expression in 94.1 ± 3% L274G-hiPSCs at passage (p) 30–34 (Supplementary Fig. S1D). Additionally, flow analysis showed the expression of the pluripotency markers OCT4 and SSEA4 in both the WT-hiPSCs (p27–38) and L274G-hiPSCs (p30–34) (Supplementary Fig. S1D).

### Assessment of trilineage differentiation potential of L274G-hiPSCs

We assessed the differentiation potential of L274G-hiPSCs as compared to the WT-hiPSCs to the three-germ lineages: ectoderm, mesoderm, and endoderm. Both the WT-hiPSCs and L274G-hiPSCs exhibited differentiation to ectoderm-derived Nestin-expressing cells, mesoderm-derived Brachyury-T (BryT)-expressing cells, and endoderm-derived SOX17-expressing cells (Fig. 1D). Additionally, the L274G-hiPSCs continued to express mCherry post-differentiation indicating stable expression of the transgene in the L274G-hiPSC-derived differentiated cells (Fig. 1D).

Furthermore, the in vivo differentiation potential of both WT-hiPSCs and L274G-hiPSCs were assessed via the teratoma formation assay (Supplementary Fig. S2A). Both hiPSC lines formed significant teratomas within six weeks in all five immunocompromised mice (Supplementary Fig. S2B). However, the diameter of L274G-hiPSC-derived teratomas was significantly smaller as compared to the teratomas derived from WT-hiPSCs (Supplementary Fig. S2C). Histology showed the presence of immature ectodermal, endodermal and mesodermal structures in the WT-hiPSC-derived teratomas that were multifocally surrounded by bands of mature mesodermal tissue (muscle) (Fig. 1E, i–iv). Multifocally, immature neuroectoderm predominated and consisted of small primitive-appearing hyperchromatic cells arranged in rosettes or tubules that resemble structures seen in the early embryonic central nervous system (Fig. 1E, i and iii). Additionally, primitive epithelium (endoderm) surrounded by bands of muscle (mesoderm) and multifocal melanin pigment was also observed in these teratomas (Fig. 1E, ii, iv). In the L274G-hiPSC-derived teratomas, we observed immature ectodermal, endodermal and mesodermal structures that were multifocally surrounded by bands of mature mesodermal tissue (muscle) (Fig. 1E, v–viii). Furthermore, like the WT-hiPSCs, immature neuroectoderm predominated in the L274G-hiPSC-derived teratomas (Fig. 1E, v, vii, and viii) as well as primitive epithelium (endoderm) surrounded by bands of muscle (mesoderm) (Fig. 1E, v, vi, and viii). Taken together, our results showed no significant morphologic differences between the differentiation potential of WT-hiPSCs and L274G-hiPSCs in vitro or in vivo.

### Characterization of Anl incorporation in L274G-hiPSC proteome

**Fig. 2:**
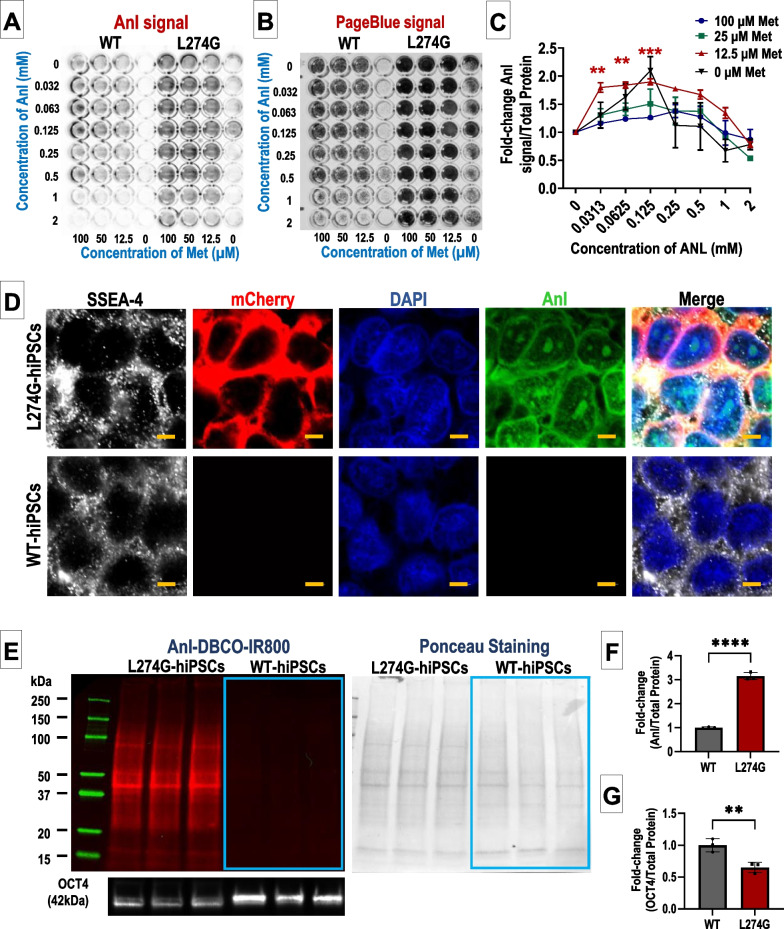
Anl tagging of proteins in L274G-hiPSCs. **A** IR800 (left) and **B** PageBlue (right) signal showing Anl-tagged and total proteins in L274G-hiPSCs cultured in varying concentrations of Anl and Met. N = 3, **p < 0.01, ***p < 0.005 compared to control (100 µM Methionine). **C** Quantification of dose-dependent Anl tagging in L274G-hiPSCs. **D** Fluorescence images showing expression of SSEA4, mCherry, and Anl tagging in L274G-hiPSCs and WT-hiPSCs. Scale bar: 5 µm. **E** Representative images showing ANL-DBCO-IR800 tagging in L274G-hiPSC proteome, the corresponding ponceau staining and OCT4 expression in hiPSCs. Absence of Anl tagging in WT-hiPSCs is highlighted by blue boxes. **F** Quantification of Anl tagging (IR800) of proteome and **G** Quantification of OCT4 expression normalized to total protein (Ponceau stain) in WT- and L274G-hiPSCs. N = 3. ****p < 0.0001; **p < 0.01. Full-length blots have been shown in supplementary figure S5

To determine the optimum concentration of Anl and methionine for non-canonical amino acid tagging without cell death, we cultured the L274G-hiPSCs in varying concentrations of methionine (0, 12.5, 25, 50, and 100 µM) and Anl (0, 0.03125, 0.0625, 0.125, 0.25, 0.5, 1, and 2 mM) for 24 h (Fig. 2A). Furthermore, to eliminate non-specific Anl incorporation, we also cultured WT-hiPSCs under similar culture conditions (Fig. 2A). After 24 h, the L274G-hiPSCs and WT-hiPSCs were click-reacted with DBCO-IR800 and the IR800 signal in each well was normalized to the total protein (determined by staining with Page-Blue) (Fig. 2B, C). We observed a dose-dependent increase in IR800 signal at lower Anl concentrations with a slight decrease in signal at high Anl concentrations (Fig. 2C). While the highest IR800/total protein signal was observed under 0 µM methionine culture conditions, complete absence of methionine appeared to be detrimental to cell survival. Furthermore, L274G-hiPSCs cultured in medium containing 12.5 µM methionine and 0.125 mM Anl had a significantly higher IR800 signal compared to cell cultured in 100 µM methionine and was optimized for non-canonical amino acid tagging of L274G-hiPSCs.

Intracellular Anl incorporation in the L274G-hiPSC proteome was validated using FUNCAT and immunocytochemistry. We observed Anl incorporation in the total proteome of L274G-hiPSCs (non-specific to cell compartment) but not in WT-hiPSCs cultured under similar conditions (Fig. 2D). On the other hand, mCherry and SSEA-4 immunofluorescence was restricted to the cytoplasm and cell membrane, respectively, in the L274G-hiPSCs (Fig. 2D). As expected, we did not observe mCherry fluorescence in the WT-hiPSCs but detected the expression of SSEA-4 in the plasma membrane (Fig. 2D). Furthermore, we validated the Anl incorporation into the total proteome of L274G-hiPSCs using BONCAT-western blot at different passages (p12, p23, and p28) (Fig. 2E). We observed Anl incorporation throughout the proteome of the L274G-hiPSCs at all three passages, evidenced by the presence of IR800 signal associated with proteins of all molecular sizes (Fig. 2E, F). Moreover, no Anl incorporation was observed in the WT-hiPSC proteome following culture of these cells in Anl-containing medium (Fig. 2E, F). However, we observed a significantly lower expression of OCT4 in the L274G-hiPSCs as compared to the WT-hiPSCs (Fig. 2G).

**Fig. 3:**
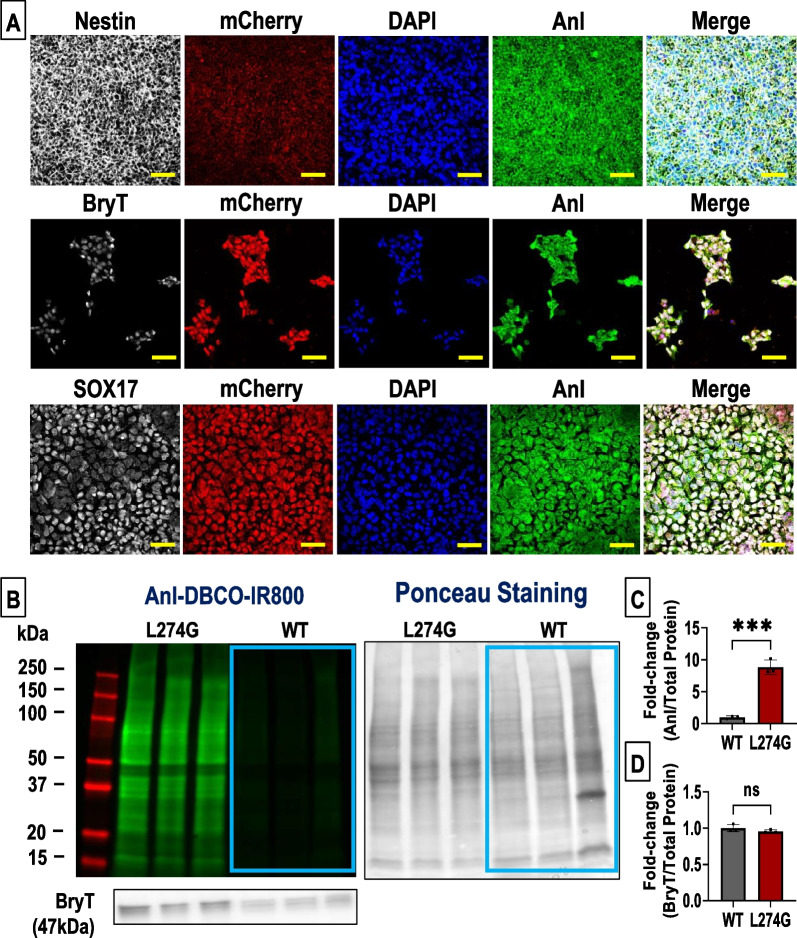
Assessment of ANL tagging in differentiated L274G-hiPSCs. **A** Fluorescence images showing expression of mCherry and Anl tagging in L274G-hiPSC-derived Nestin-expressing ectodermal cells, BryT-expressing mesodermal cells, and SOX17-expressing endodermal cells. Scale bar: 50 µm. **B** Representative images showing Anl-DBCO-IR800 tagging of proteome, the corresponding ponceau staining and BryT expression in WT-and L274G-hiPSC-derived mesodermal cells. Absence of Anl tagging in WT cells is highlighted by blue boxes. **C** Quantification of Anl tagging (IR800) of proteome and **D** Quantification of BryT expression normalized to total protein (Ponceau stain) in WT- and L274G-hiPSC-derived mesodermal cells. N = 3 from three independent cultures of different passages. ***p < 0.001. Full-length blots have been shown in supplementary figure S5

We next assessed the incorporation of Anl into L274G-derived differentiated cell proteome. WT-hiPSC and L274G-hiPSC-derived ectoderm, endoderm and mesoderm cells were cultured in 12.5 µM methionine and 0.125 mM Anl-supplemented medium for 24 h. FUNCAT analysis showed the incorporation of Anl into Nestin-expressing ectodermal cells, Bry-T-expressing mesodermal cells, and SOX17-expressing endodermal cells (Fig. 3A) differentiated from L274G-hiPSCs. Furthermore, we observed the presence of Anl-DBCO-IR800 signal in the total proteome in the L274G-hiPSC-derived differentiated cells but not in the WT-hiPSCs following mesoderm (Fig. 3B, C), ectoderm (Supplementary Fig. S3A, B) and endoderm (Supplementary Fig. S3D, E) differentiation. Furthermore, we did not observe any significant changes in the expression of lineage-specific markers, BryT (mesoderm) (Fig. 3B, D), Nestin (ectoderm) (Supplementary Fig. S3A, C) and SOX17 (endoderm) (Supplementary Fig. S3D, F) between the L274G-hiPSC- and WT-hiPSC-derived differentiated cells. Taken together, our data showed non-canonical amino acid tagging in the cellular proteome of L274G-hiPSCs and their derived differentiated cells with no significant changes in cellular protein synthesis.

### Cell-specific Anl incorporation in L274G-hiPSC-CMs

**Fig. 4:**
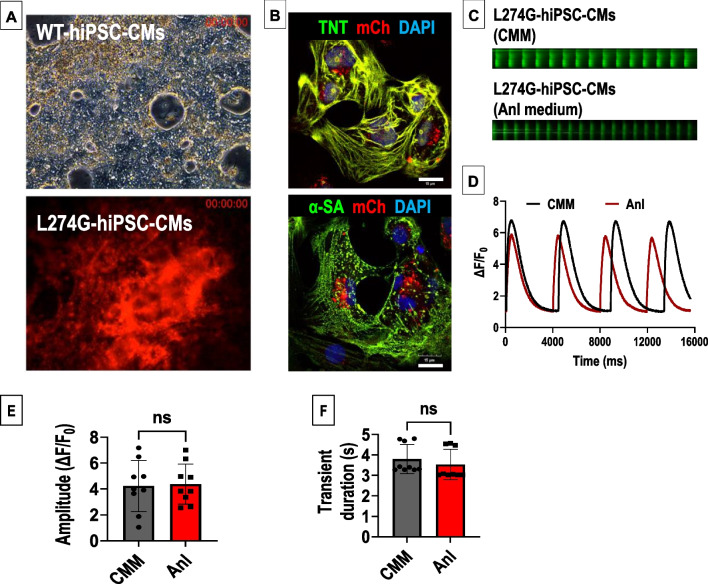
Assessment of Anl-tagging in L274G-hiPSC-CMs. **A** Representative images showing hiPSC-CMs differentiated from WT-hiPSCs and L274G-hiPSCs. Images correspond to supplemental videos V1 and V2, respectively. **B** Confocal images showing expression of TNT and α-SA in L274G-hiPSC-CMs. **C**–**D** Representative traces showing **C** calcium transients and **D** their quantification in L274G-hiPSC-CMs cultured in CMM and Anl-supplemented culture medium. Quantitative assessment of calcium transient amplitude **E** and duration **F** in L274G-hiPSC-CMs cultured in CMM and Anl-supplemented medium. N = 9 cells from three independent cultures. Data shown is an average of three calcium cycles per cell

Both WT-hiPSCs and L274G-hiPSCs efficiently differentiated to function hiPSC-CMs within 13 days of differentiation (Fig. 4A, Supplemental Video 1 and Supplemental Video 2). Immunocytochemical analysis showed the expression of mCherry as well as cardiomyocyte-specific markers, troponin-T (TNT) and α-sarcomeric actinin (α-SA) in the L274G-hiPSC-CMs (Fig. 4B). Furthermore, following culture of L274G-hiPSC-CMs in Anl-supplemented medium, we observed small but insignificant changes in contractility of the cells as compared to L274G-hiPSC-CMs cultured in CMM, evident from their intracellular calcium transients (Fig. 4C–F).

**Fig. 5:**
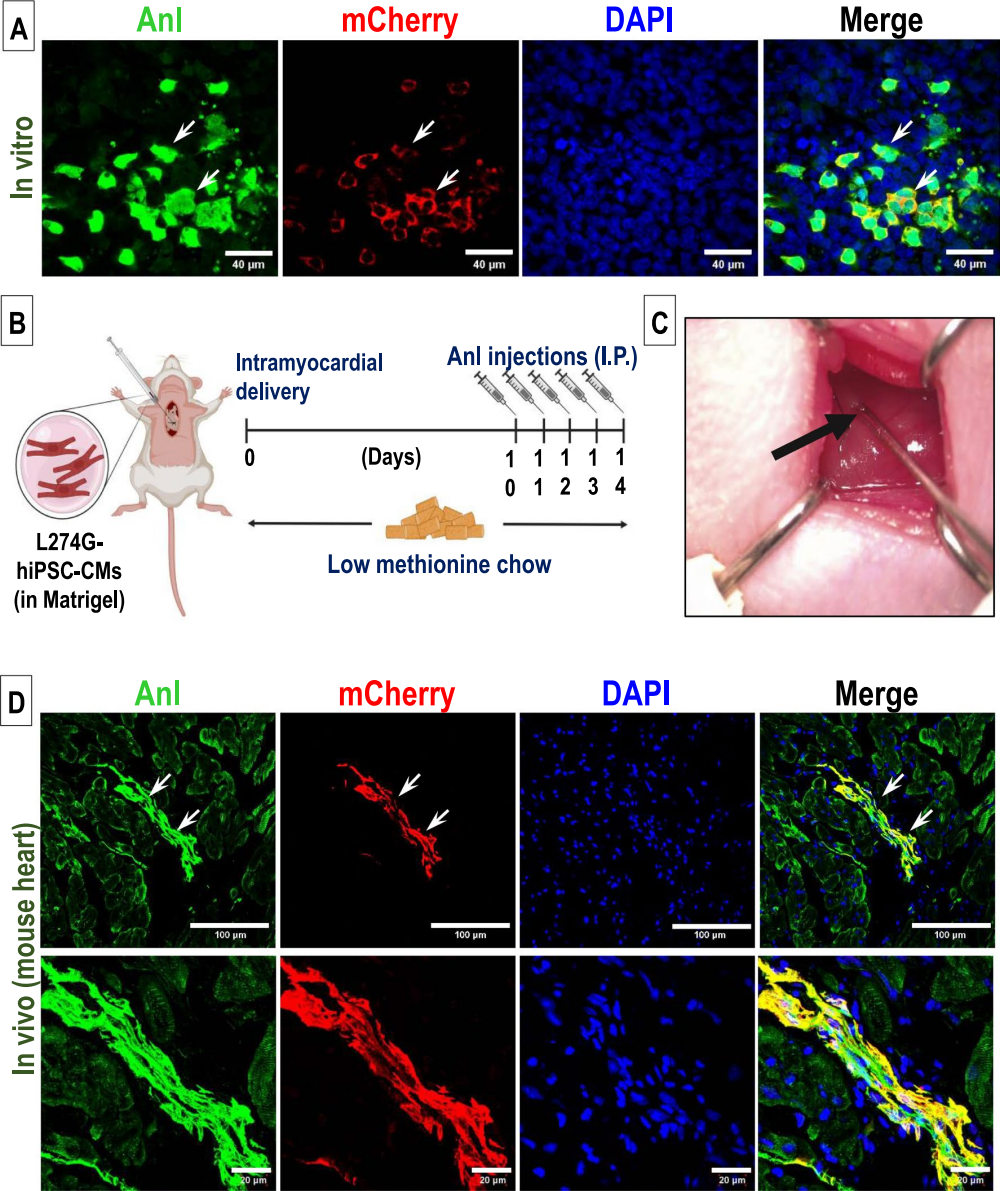
Assessment of cell-specific Anl-tagging in L274G-hiPSC-CMs. **A** Fluorescence images showing expression of DBCO-FITC and mCherry expression in L274G-hiPSC-CMs co-cultured with WT-HUVECs. Scale bar: 40 µm **B** Schematic showing experimental design for transplantation of L274G-hiPSC-CMs in mouse hearts. **C** Representative image showing intramyocardial injection (arrow) of L274G-hiPSC-CMs. **D** Fluorescence images showing expression of DBCO-FITC and mCherry expression in L274G-hiPSC-CMs in mouse cardiac tissue sections two weeks post-transplantation. Scale bar: Top panel: 100 µm, Bottom panel: 20 µm

To determine if Anl incorporation into cell proteome was specific to L274G-hiPSC-derived cells, we co-cultured the differentiated L274G-hiPSC-CMs with WT-HUVECs for 24 h in 12.5 µM methionine and 0.125 mM Anl supplemented medium. Following FUNCAT and immunofluorescence staining, we observed DBCO-FITC fluorescence only in the mCherry^+^ L274G-hiPSC-CMs (Fig. 5A). These observations validated the selective incorporation of Anl in L274G-hiPSC-CM proteome. Furthermore, to validate the cell-specific Anl-tagging in vivo, we transplanted L274G-hiPSC-CMs intramyocardially in athymic nude mice (Fig. 5B, C). No significant weight differences were observed in the mice fed with low methionine diet during the study. In the cell transplant group, immunohistochemical analysis of cardiac sections showed engrafted L274G-hiPSC-CMs which were identified by their mCherry fluorescence (Fig. 5C). Furthermore, FUNCAT showed Anl incorporation only in the mCherry^+^ transplanted L274G-hiPSC-CM proteome and not in the surrounding wild-type host tissue (Fig. 5D). Furthermore, we validated our observation via western blot analysis of click reacted lysates from left ventricle (LV), lung, kidney and liver of control mice and mice transplanted with L274G-hiPSC-CMs. Our data showed presence of Anl tagged proteins only in the LV lysates of mice from the cell transplantation group but not in other tissues (Supplementary Fig. S4A). Similarly, we observed no Anl incorporation in control mice which received Anl injections but no cell transplantation (Supplementary Fig. S4A).

**Fig. 6:**
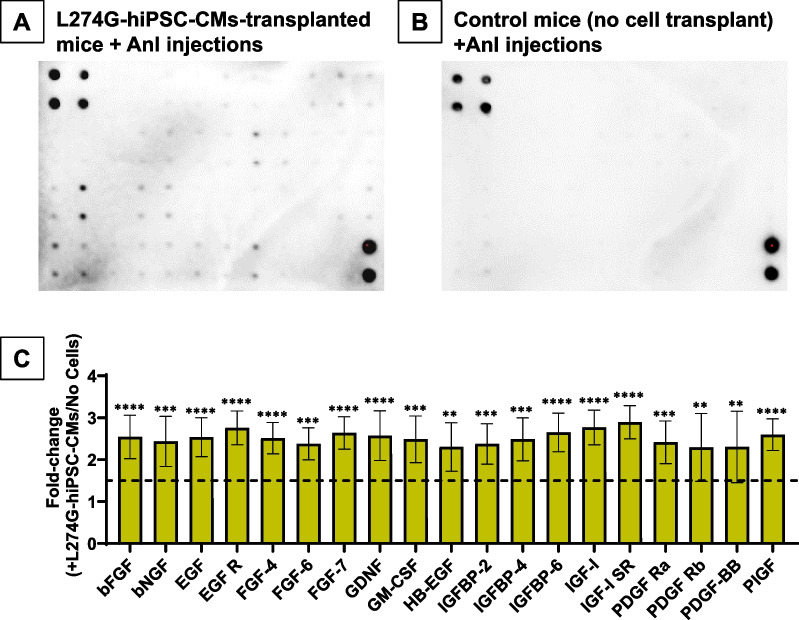
Detection of transplanted L274G-hiPSC-CM-specific secreted proteins in mouse plasma. Representative protein array showing presence/absence of 41 Anl-tagged proteins in plasma of L274G-hiPSC-CMs-transplanted mice (**A**) and control mice (**B**). **C** Quantification of 20 growth factors in L274G-hiPSC-CM treated mice plasma normalized to control mice. N = 3 mice/group. Enrichment cut-off: 1.5-fold (dashed line). **p < 0.005; ***p < 0005; ****p < 0.0001

Furthermore, we assessed the presence of L274G-hiPSC-CM-specific Anl-tagged proteins in the mouse plasma using protein arrays (Fig. 6; Supplementary Fig. S4B). We compared the plasma from L274G-hiPSC-CM-transplanted mice with plasma from control mice. Our data showed a positive detection of Anl tagged proteins only in the plasma of mice which were transplanted with L274G-hiPSC-CMs (Fig. 6A) but not in plasma of control mice (Fig. 6B). Since both mice groups were given daily injections of Anl, our data confirmed negligible non-specific incorporation of Anl in WT host tissue proteome. Furthermore, quantitative analysis of the protein arrays showed a significant enrichment (> 1.5 fold) of 20 out of 41 proteins in the L274G-hiPSC-CM-transplanted mouse plasma as compared to control mice (Fig. 6C) indicating secretion of these proteins by the transplanted L274G-hiPSC-CMs.

Taken together, our results showed cell-specific Anl incorporation only in the L274G-hiPSC-CM proteome both in vitro and in vivo.

## Discussion

In the present study, we have developed the transgenic L274G-hiPSC line expressing the L274G gene to identify cell-specific proteome in hiPSC-derived differentiated cells via BONCAT. We also demonstrated the ability of L274G-hiPSCs to efficiently differentiate into cells of all the three germ lineages both in vitro as well as in vivo. Furthermore, we established L274G-hiPSC-specific incorporation of Anl into the proteome both in a co-culture system (in vitro) and post-transplantation (in vivo). Additionally, we were able to detect the presence of Anl-tagged L274G-hiPSC-CM-specific proteins in the plasma of mice two weeks post-transplantation in healthy mouse hearts. Therefore, our findings establish the applicability of this novel hiPSC line to track transplanted hiPSC-derived differentiated cell-specific proteome and gain insights into the paracrine mechanisms underlying hiPSC-based cell therapies.

Since their discovery, hiPSCs have provided a beacon of hope for the development of cell-based approaches in regenerative medicine. hiPSC-derived differentiated cells have been tested for treating a myriad of diseases including Parkinson’s disease, spinal cord injury, diabetes mellitus, macular degeneration, myocardial infarction, and muscular dystrophy [28–30]. However, emerging reports from preclinical and clinical studies have shown that contrary to the hypothesis that transplanted hiPSC-derived differentiated cells would replace damaged cells and tissues, the beneficial outcomes observed in these studies are paracrine in nature [12–14]. Therefore, understanding the secretome of transplanted hiPSCs could provide key mechanistic information for designing and developing future therapeutic strategies for regenerative medicine.

In vitro studies have evaluated the composition of hiPSC-derived differentiated cell secretome in single cell-type cultures. However, there is a paucity in knowledge pertaining to the proteome or secretome of cells in a complex multicellular environment like in vitro co-culture systems or in vivo tissue microenvironment. Several methods like stable isotope labeling using amino acids in cell culture (SILAC) [31, 32], isobaric tag for relative and absolute quantitation (iTRAQ) [33, 34], and BONCAT using azidohomoalanine or homopropargylglycine have been developed for metabolic labelling of cell proteome [35–37]. However, each of these methods has its advantages and disadvantages. For *e.g.* SILAC requires culturing cells in radioisotope-supplemented medium for several generations and none of these techniques can identify cell-specific proteome in co-culture systems [31–36]. On the other hand, a previous study had shown the identification of Anl-tagged bacteria-derived secreted proteins in the infected mammalian host cells in vitro, using BONCAT [38]. Therefore, in our study, we expressed the transgene for mutant mouse methionyl tRNA synthetase, L274G, in hiPSCs. L274G expression enabled incorporation of Anl in place of methionine in the newly synthesized nascent proteins of L274G-hiPSCs and their derived differentiated cells, but not WT cells. Therefore, we were able to successfully detect Anl incorporation only in L274G-hiPSC-CMs both in vitro when co-cultured with WT-HUVECs as well as in vivo post-transplantation into mouse hearts. Our results are consistent with previous reports which showed selective incorporation of Anl into L274G-expressing cells in in vitro co-culture systems [25] or in vivo [39, 40].

In the present study, we were able to detect > 1.5-fold enrichment of 20 different Anl-tagged growth factors in the plasma of mice up to two weeks post-transplantation with L274G-hiPSC-CMs compared to control mice. Our observations are consistent with previous in vitro studies that have shown the presence of these growth factors in hiPSC-CM secretome [41]. Among the identified growth factors, bFGF, PDGF, EGF, HB-EGF and IGF-1, have been shown to promote angiogenesis and cardioprotection [42]. Therefore, our observations are consistent with previous studies that have reported improved angiogenesis and decreased apoptosis following hiPSC-CM transplantation [43]. Furthermore, since we observed negligible non-specific signals in the plasma or tissues of control mice which were given Anl injections for similar durations, our data strongly suggests the transplanted L274G-hiPSC-CMs as the source of these Anl-tagged proteins. Additionally, considering the significantly small ratio of transplant-derived proteins (from ~ 2.5 million transplanted hiPSC-CMs) to host-derived proteins in the plasma, the positive detection of Anl-tagged proteins establishes the robustness of our BONCAT system. Taken together, our study, for the first time, establishes a proof-of-concept of the applicability of cell-specific metabolic labelling to identify the in vivo secretome profile of transplanted L274G-hiPSC-derived differentiated cells.

Additionally, the L274G-hiPSCs exhibited all characteristic properties of a typical hiPSC line including the expression of the pluripotency markers, OCT4, SOX2, SSEA-4 and TRA-1-60. The L274G-hiPSCs also exhibited efficient differentiation to all three germ lineages: ectoderm, mesoderm, and endoderm in vitro and were comparable with the WT-hiPSCs. Moreover, the expression of the tested protein levels in the differentiated cells was comparable between the L274G-hiPSCs and WT-hiPSCs. Our observation is consistent with previous studies have shown that Anl incorporation does not grossly affect the abundance or content of the proteome in multiple cell types [18, 44, 45]. However, we did observe a significantly lower expression of OCT4 in the L274G-hiPSCs as compared to the WT-hiPSCs. Moreover, although both the WT-hiPSCs and L274G-hiPSCs could form teratomas having similar differentiated cell types in vivo, the teratomas derived from L274G-hiPSCs were significantly smaller than those derived from the WT-hiPSCs. It is possible that the introduction of the L274G transgene via lentivirus-based transduction may have affected the OCT4 expression and proliferation rate of the L274G-hiPSCs via introduction of spontaneous mutations [46]. However, there are several studies that have shown a significant influence of cell line, passage number and site of injection on the teratoma size [47, 48]. Since both the cell lines showed comparable trilineage differentiation potential both in vitro and in vivo, we believe that the L274G-hiPSC line does not carry significant mutations which impact their pluripotency.

We observed a small but insignificant change in L274G-hiPSC-CM contractility following culture in methionine-reduced, Anl-supplemented CMM as compared to L274G-hiPSC-CMs cultured in regular CMM. Previous reports indicated small changes in the metabolic profile of *Escherichia coli* (E.coli) cells cultured in the presence of non-canonical amino acids [49]. This study showed an alteration in TCA-cycle associated metabolites which may impact mitochondrial function. However, unlike the *E. coli* cells, the changes observed in our culture system were not significant. Therefore, future studies would have to be carried out to evaluate the effect of non-canonical amino acid incorporation on L274G-hiPSC-CM function.

## Conclusions and future directions

Overall, our study demonstrated for the first time that L274G-hiPSCs can be used to study the cell-specific proteome in vitro as well as in vivo and will pave the way for understanding cell specific responses to external stimuli, like tissue microenvironment, inflammation, and ischemic stress. Furthermore, since the L274G-hiPSCs can be differentiated to a plethora of cell types which have been used for tissue regeneration, including neurons, skeletal muscle cells, pancreatic β-cells, hepatocytes, and endothelial cells, these cells can be used to identify the transplanted cell-specific proteomic responses in different disease models. However, some key aspects including (a) concentration and half-life of the secreted proteins, (b) tissue/cell of origin, (c) transplanted cell-type, (d) underlying disease pathology, (e) number of cells transplanted, and (f) survival and retention of the transplanted cells will have to be optimized to establish the transplanted cell-specific secretome profile in future studies.

### Supplementary Information

Additional file 1 

Additional file 2 

Additional file 3 

## Data Availability

The datasets generated and/or analyzed during the current study are available from the corresponding author on reasonable request.

## Abbreviations

Anl: Azidonorleucine
BONCAT: Bioorthogonal non-canonical amino acid tagging
CDM: Cardiomyocyte differentiation medium
CMM: Cardiomyocyte maintenance medium
DBCO: Dibenzocyclooctyne
FUNCAT: Fluorescent non-canonical amino acid tagging
hiPSC: Human induced pluripotent stem cells
hiPSC-CM: Human induced pluripotent stem cell derived cardiomyocytes
HUVECs: Human umbilical vein endothelial cells
IAA: Iodoacetamide
iTRAQ: Isobaric tag for relative and absolute quantitation
L274G: L274G*Mm*MetRS
SILAC: Stable isotope labeling using amino acids in cell culture
WT: Wild type
