# Supplementary material for: Bioorthogonal non-canonical amino acid tagging to track transplanted human induced pluripotent stem cell-specific proteome
Source: Stem Cell Res Ther. 2024 Jun 26;15:186. doi: 10.1186/s13287-024-03792-3 (PMC11210150; doi:10.1186/s13287-024-03792-3)

**SUPPLEMENTARY DATA**

**Supp Fig. S1. In vitro characterization of L274G-hiPSCs. (A)** Phase-contrast and fluorescence microscopy images of L274G-hiPSC and WT-hiPSC colonies showing typical colony morphology and mCherry expression, respectively. Scale bar: 100 µm. **(B)** Immunostaining for PSC markers, SOX2 and TRA-1-60 in L274G-hiPSCs. Scale bar: 20 µm. **(C)** Immunostaining for PSC markers, OCT4, SSEA4, SOX2 and TRA-1-60 in WT-hiPSCs. Scale bar: 20 µm. **(D)** Flow cytometry showing percentage of mCherry^+^, OCT4^+^ and SSEA4^+^ cells in L274G-hiPSCs and WT-hiPSCs. N=3 independent cultures.

**Supp Fig. S2. Assessment of trilineage differentiation of L274G-hiPSCs. (A)** Experimental design to assess in vivo differentiation potential of hiPSCs using teratoma formation assay. **(B)** Representative image showing teratoma formed in mice after 6 weeks. **(C)** Measurement of teratomas derived from L274G-hiPSCs and WT-hiPSCs after 6 weeks. N=5, *:p<0.05.

**Supp. Fig. S3. Assessment of ANL tagging in differentiated L274G-hiPSCs. (A)** Representative images showing ANL-DBCO-IR800 tagging of proteome, the corresponding ponceau staining and Nestin expression in WT-and L274G-hiPSC-derived ectodermal cells. **(B)** Quantification of Anl tagging (IR800) of proteome and **(C)** Quantification of Nestin expression normalized to total protein (Ponceau stain) in WT- and L274G-hiPSC-derived ectodermal cells. **(D)** Representative images showing ANL-DBCO-IR800 tagging of proteome, the corresponding ponceau staining and SOX17 expression in WT-and L274G-hiPSC-derived endodermal cells. **(E)** Quantification of Anl tagging (IR800) of proteome and **(F)** Quantification of SOX17 expression normalized to total protein (Ponceau stain) in WT- and L274G-hiPSC-derived endodermal cells. Absence of Anl tagging in WT cells is highlighted by blue boxes in **(A)** and **(D)**. N=3 from three independent cultures of different passages. ****:p<0.0001. Full-length blots are shown in supplementary figure S5.

**Supp. Fig. S4. Assessment of intracellular Anl tagging in L274G-hiPSC-CMs in vivo**. **(A)** Representative images of blot showing presence or absence of Anl-tagged proteins in the left ventricle of the heart (LV), lungs, liver, and kidneys of mouse transplanted with L274G-hiPSC-CMs (L) and control mouse (C) and the corresponding ponceau staining. **(B)** Table showing the array map for the Human Growth factor Array C1.

**Supp. Fig. S5. Full-length blots shown in the study**. Images shown in **(A-B), (C-D), (E-F),** and **(G-H)** correspond to cropped blots shown in Fig 2(E), 3(B), S3(A), and S3(D), respectively. Cropped regions are highlighted by yellow dotted boxes in the full-length blots.

**SVideo 1: Videomicrography showing contractility in WT-hiPSC-CMs**

**SVideo 2: Videomicrography showing contractility in WT-hiPSC-CMs**


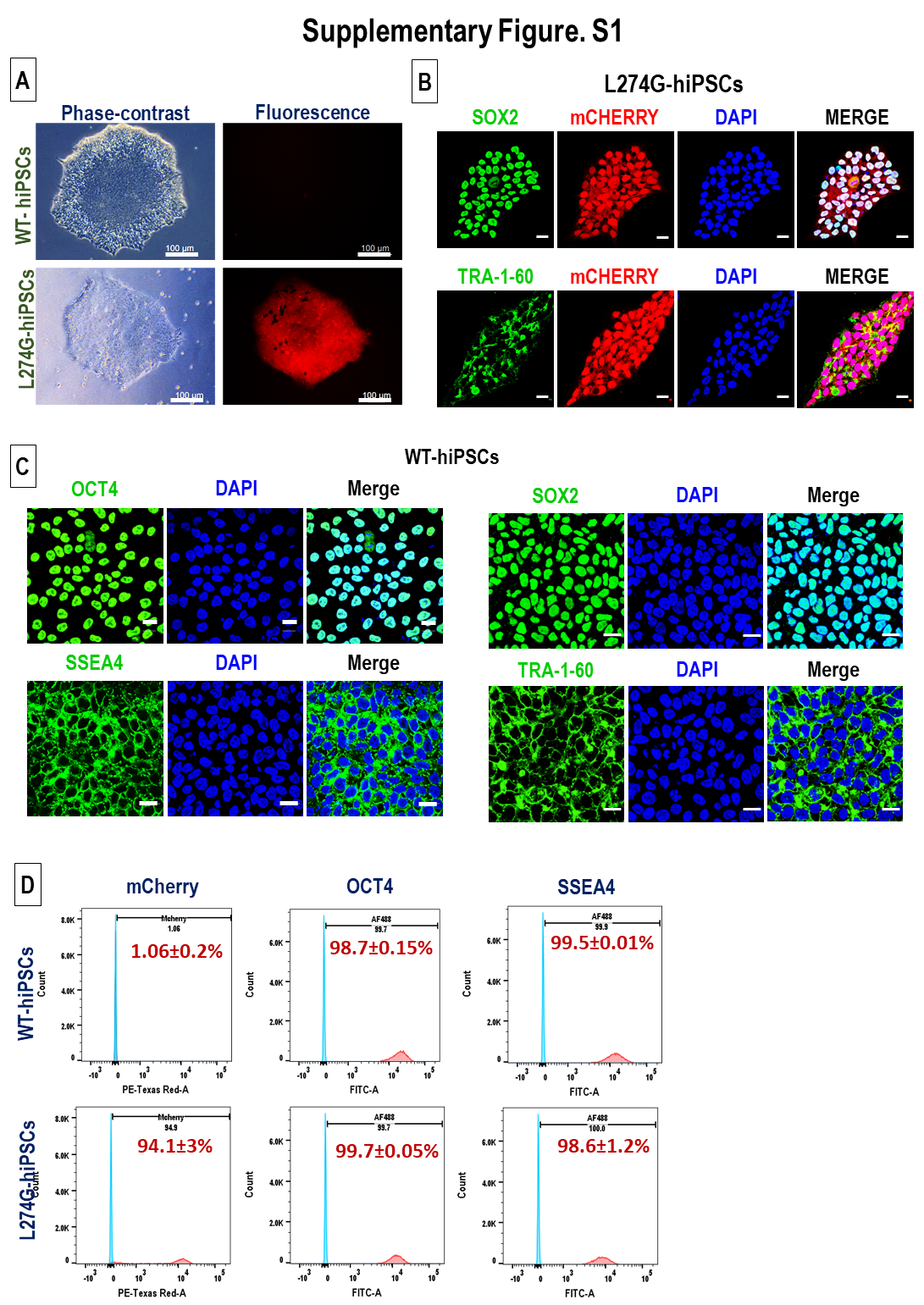


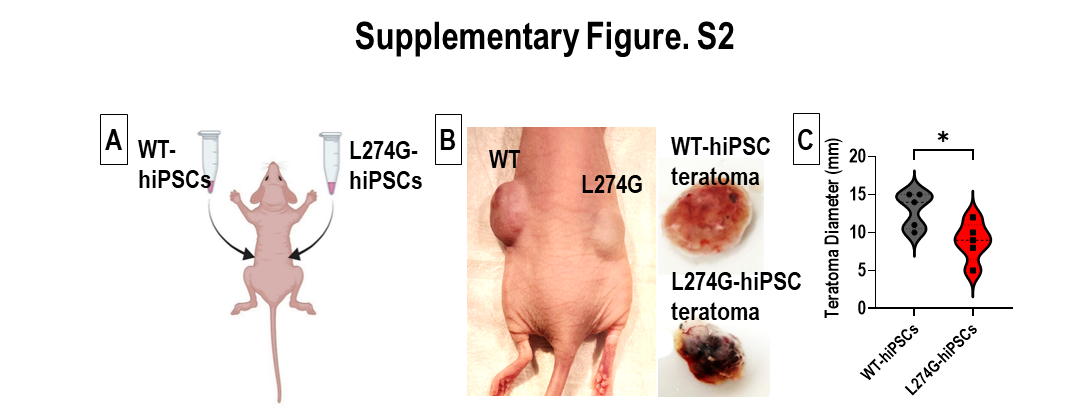


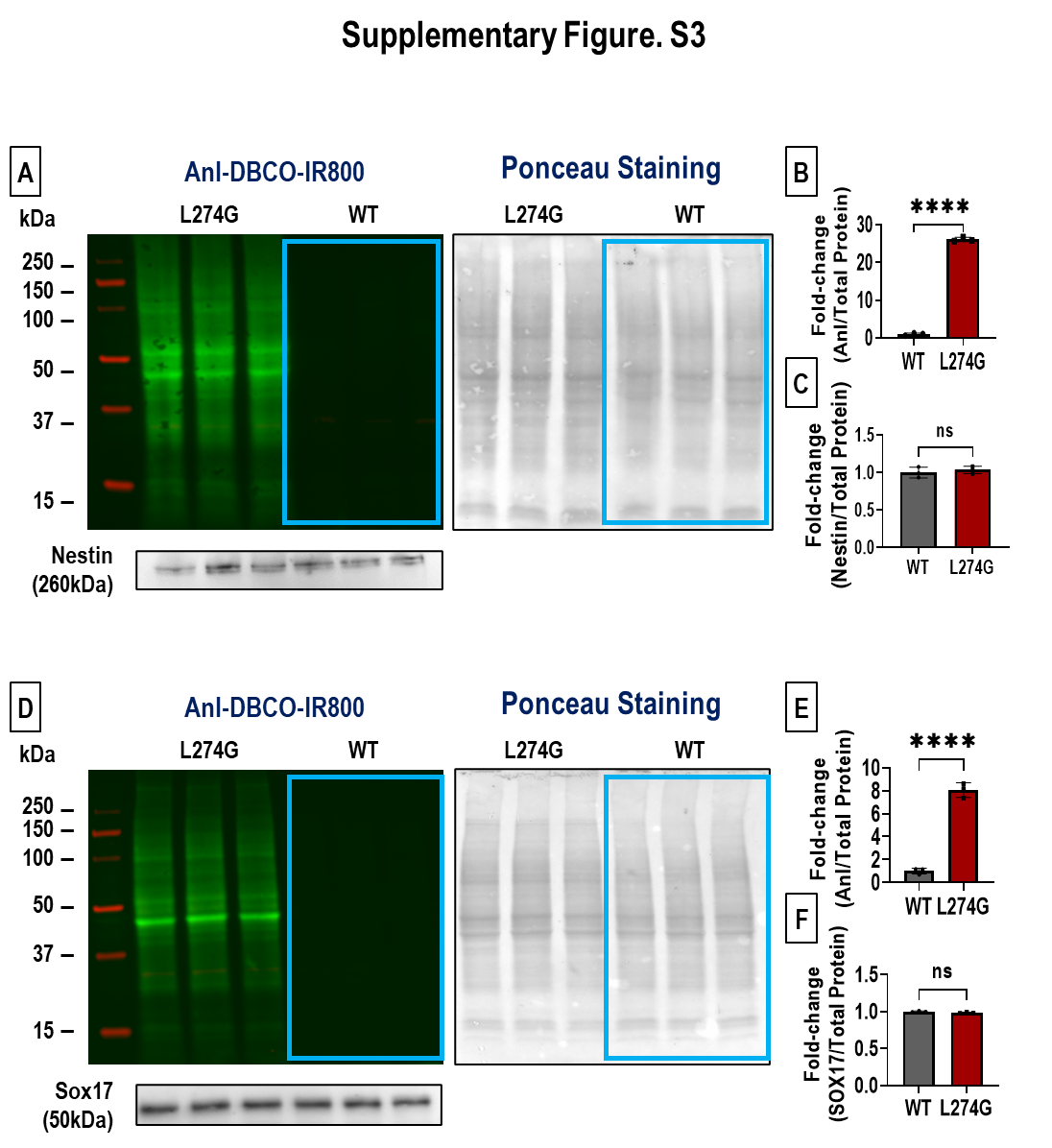


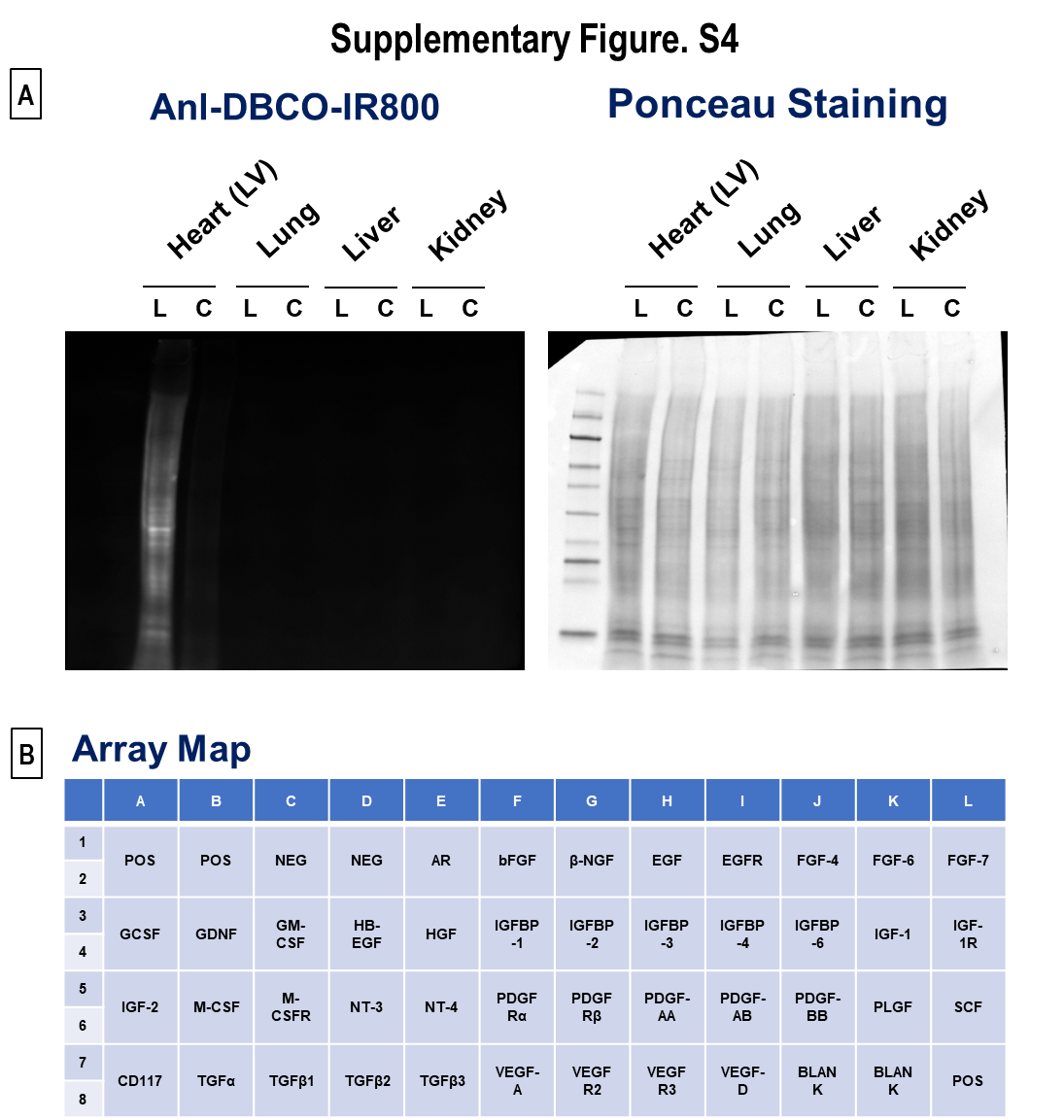


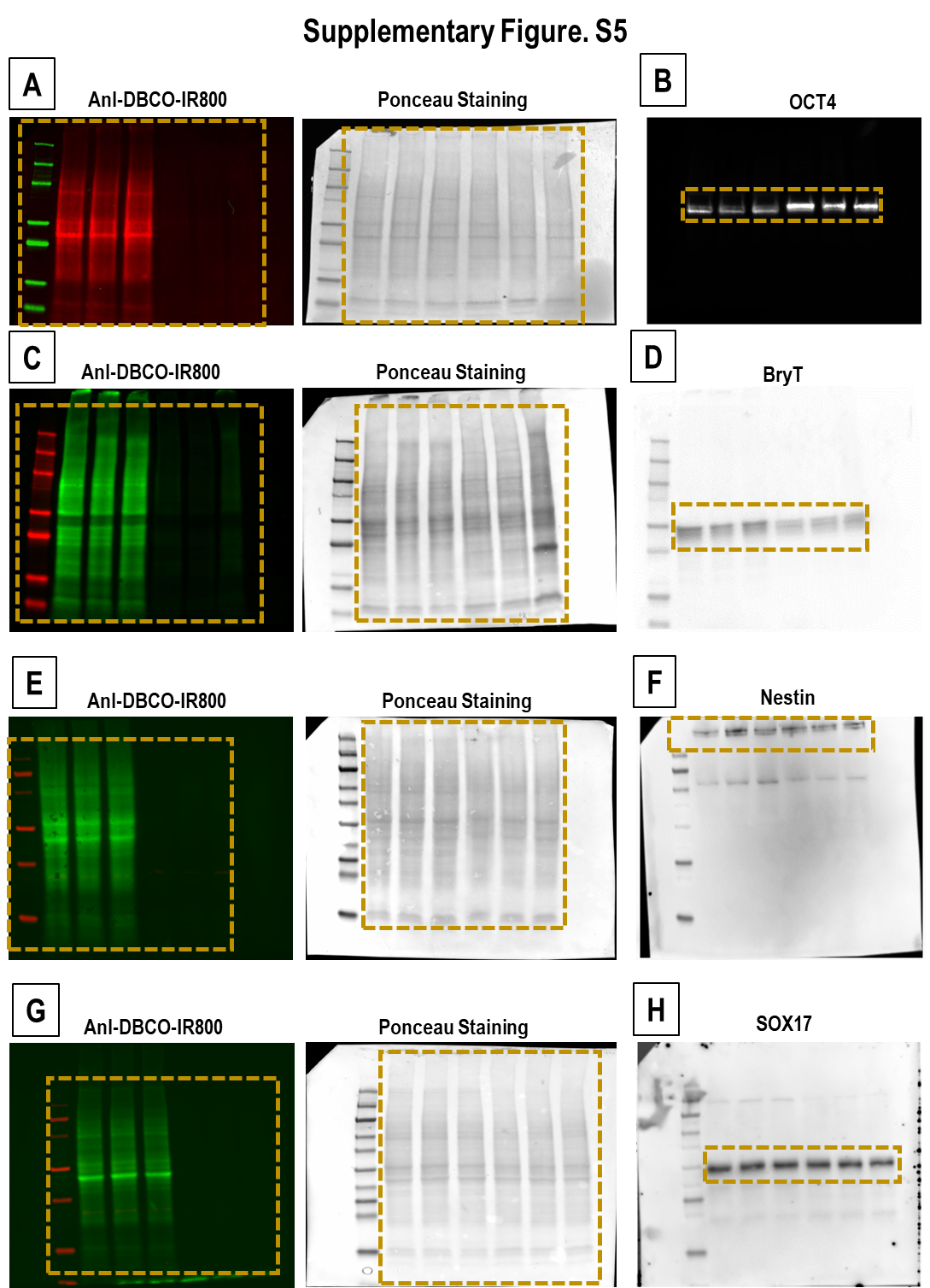

Supplement: Supplementary file 3 — Additional file 3 [file 13287_2024_3792_MOESM3_ESM.docx]
